# An 80 years old woman with acute gastric volvulus

**DOI:** 10.11604/pamj.2021.38.12.25391

**Published:** 2021-01-06

**Authors:** Danilo Coco, Silvana Leanza

**Affiliations:** 1 Department of General Surgery, Ospedali Riuniti Marche Nord, Pesaro, Italy,; 2 Department of General Surgery, Carlo Urbani Hospital, Jesi, Ancona, Italy

**Keywords:** Gastric volvulus, thymoma, tumor

## Image in medicine

Gastric volvulus is an abnormal rotation of the stomach around itself. It is a life-threatening condition due to the risk of abs ingestis pneumonia or strangulation and gangrene/perforation of the stomach. Imaging as X-ray transit of the first digestive tract or Thoracic CT Scan is usually diagnostic. The population affected ranges from pediatric age group to elderly with multiple co-morbidities. The mortality from acute gastric volvulus is now 15%-20%. An 80-year-old Caucasian man was admitted to Emergency Room of our institution for dysphagia, vomiting and epigastric pain. His past medical history was negative for previous gastrointestinal disease or surgery. He referred only dysphagia for solid food and mild retrosternal pain. On physical examination, the patient was in slight distress. Oxygen saturation on pulse oximetry was 85%, blood pressure (BP) was 90/75 mmHg while supine, heart rate was 100/min and the temperature was 36°C. On auscultation, he had breath sounds were bilaterally reduced. His abdominal physical examination was unremarkable. The abdomen was soft, non-tender, mildly distended. White blood tests were in normal range. Computed tomography (CT) scan showed the displacement of the stomach above the gastro esophageal junction; the stomach appeared upside-down with the antrum showing huge gastric distension with no free fluid or air. We performed an explorative laparoscopy which confirmed the intra-thoracic rotation of the stomach. We proceed to release it from its adherence from the thorax to the abdomen and complete the operation with a laparoscopic Nissen fundoplication. Postoperative recovery was uneventful. Esophagus transit was normal. At the 3-day, the patient was in good clinical condition and he was discharged.

**Figure 1:**
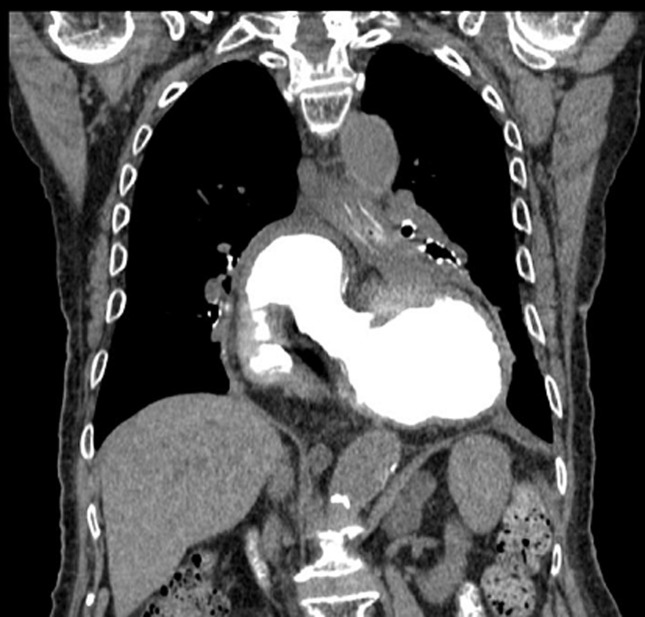
computed tomography (CT) scan showed the displacement of the stomach above the gastro esophageal junction; the stomach appeared upside-down with the antrum showing huge gastric distension with no free fluid or air

